# Genetic defects of *CHM* and visual acuity outcome in 24 choroideremia patients from 16 Japanese families

**DOI:** 10.1038/s41598-020-72623-1

**Published:** 2020-09-28

**Authors:** Takaaki Hayashi, Shuhei Kameya, Kei Mizobuchi, Daiki Kubota, Sachiko Kikuchi, Kazutoshi Yoshitake, Atsushi Mizota, Akira Murakami, Takeshi Iwata, Tadashi Nakano

**Affiliations:** 1 Department of Ophthalmology, Katsushika Medical Center, The Jikei University School of Medicine, 6-41-2 Aoto, Katsushika-ku, Tokyo, 125-8506 Japan; 2 Department of Ophthalmology, The Jikei University School of Medicine, Tokyo, Japan; 3 Department of Ophthalmology, Nippon Medical School, Chiba Hokusoh Hospital, Chiba, Japan; 4 Division of Molecular and Cellular Biology, National Institute of Sensory Organs, National Hospital Organization, Tokyo Medical Center, Tokyo, Japan; 5 Department of Ophthalmology, Teikyo University School of Medicine, Tokyo, Japan; 6 Department of Ophthalmology, Juntendo University, Faculty of Medicine, Tokyo, Japan

**Keywords:** Genetics, Medical research

## Abstract

Choroideremia (CHM) is an incurable progressive chorioretinal dystrophy. Little is known about the natural disease course of visual acuity in the Japanese population. We aimed to investigate the genetic spectrum of the *CHM* gene and visual acuity outcomes in 24 CHM patients from 16 Japanese families. We measured decimal best-corrected visual acuity (BCVA) at presentation and follow-up, converted to logMAR units for statistical analysis. Sanger and/or whole-exome sequencing were performed to identify pathogenic *CHM* variants/deletions. The median age at presentation was 37.0 years (range, 5–76 years). The mean follow-up interval was 8.2 years. BCVA of the better-seeing eye at presentation was significantly worsened with increasing age (r = 0.515, p < 0.01), with a high rate of BCVA decline in patients > 40 years old. A Kaplan–Meier survival curve suggested that a BCVA of Snellen equivalent 20/40 at follow-up remains until the fifties. Fourteen pathogenic variants, 6 of which were novel [c.49 + 5G > A, c.116 + 5G > A, p.(Gly176Glu, Glu177Ter), p.Tyr531Ter, an exon 2 deletion, and a 5.0-Mb deletion], were identified in 15 families. No variant was found in one family only. Our BCVA outcome data are useful for predicting visual prognosis and determining the timing of intervention in Japanese patients with *CHM* variants.

## Introduction

Choroideremia (CHM, OMIM: #303100) is an X-linked recessive disorder that leads to progressive degeneration of the choriocapillaris, retinal pigment epithelium (RPE), and photoreceptors. CHM is caused by sequence variants or deletions in the *CHM* gene (OMIM: *300390) encoding Rab escort protein 1 (REP1)^1–4^, which is essential for intracellular vesicular trafficking^5^. The *CHM* gene is located on the X chromosome (Xq21.2), and has 15 coding exons that span a genomic region of approximately 150 kb^1,6,7^. Although REP1 is ubiquitously expressed in human tissues, CHM appears to affect primarily the RPE. In patients with CHM, loss of vision progresses from night blindness in childhood to visual field constriction in early adulthood and ultimately to legal blindness.

As of now, no treatment is available for CHM, but *CHM* gene therapy could be a promising treatment option because the only cause of CHM is the loss of functional REP1. In fact, the first human clinical trial for *CHM* gene supplementation therapy was achieved in 2014 using the adeno-associated virus 2 (AAV2) vector encoding REP1 (NSR-REP1; Nightstar Therapeutics, London, UK) in 6 patients with *CHM* variants^8^. The follow-up interval was 3.5 years, revealing that visual acuity had increased by 18–21 letters in both treated eyes of two patients^9^. Subsequently, three independent phase 2 clinical trials (24-month results) in Germany (NCT02671539)^10^, United States (NCT02553135)^11^, and Canada (NCT02077361)^12^ were performed using the NSR-REP1. All three studies yielded a similar result that baseline BCVA was generally maintained in the treated eyes.

To date, only two cohort studies from 1999 have been published of Japanese CHM patients with *CHM* variants^13,14^. In other words, a large cohort study of CHM has not been reported for the Japanese population since 1999.

Determination of the genetic background is required for upcoming phase 3 clinical trials for NSR-REP1 gene therapy in the Japanese population. Here, we aimed to investigate the genetic spectrum of *CHM* and visual acuity outcomes in a single-center cohort of Japanese patients with CHM.

## Results

### Patient characteristics

**Figure 1:**
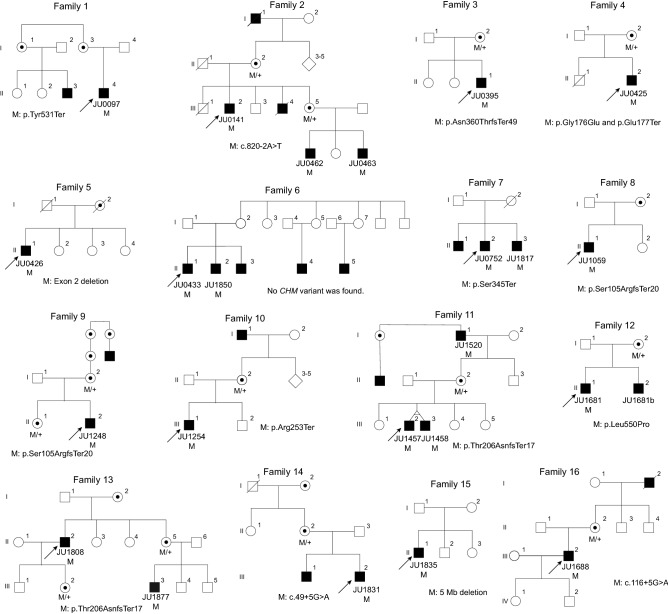
Pedigrees of 16 Japanese families with choroideremia. The proband is indicated by an arrow in each family. Affected males are indicated by solid squares and carrier females by a circle with a dot. Unaffected males and females are indicated by open squares and circles. The slash symbol indicates deceased individuals. M: mutation/pathogenic variants.

In total, 24 CHM patients from 16 unrelated Japanese families were studied (Fig. 1). Clinical findings are summarized in Table 1. All 24 patients were male. The median age at presentation was 37.0 years (range, 5–76 years), whereas the median age at follow-up was 45.5 years (range, 9–78 years). The mean follow-up interval was 8.2 years (range, 0–24 years). Four patients (16.7%) were evaluated once. Eight patients (33.3%) presented before 21 years old. Eleven patients (45.8%) presented after 40 years old.

**Table 1:** Clinical findings of 24 patients with choroideremia.

Patient #	Patient ID	Family #	Age (years) at presentation	Chief complaint	BCVA at presentation		Follow-up interval (years)	BCVA at follow up		Notes
					Decimal (RE/LE)	LogMAR (RE/LE)		Decimal (RE/LE)	LogMAR (RE/LE)	
1	JU0097	1	35	NB	1.2/–	− 0.08/–	18.0	0.9/–	0.05/–	
2	JU0141	2	42	NB, VFD	1.0/0.8	0/0.1	20.7	0.6/0.9	0.22/0.05	
3	JU0462	2	8	NB	1.2/1.0	− 0.08/0	15.4	1.2/0.9	− 0.08/0.05	
4	JU0463	2	10	NB, photophobia	1.2/1.2	− 0.08/− 0.08	9.2	1.2/1.2	− 0.08/− 0.08	
5	JU0395	3	21	Photophobia	1.2/1.2	− 0.08/− 0.08	23.8	0.7/1.2	0.15/− 0.08	
6	JU0425	4	45	NB	0.8/0.5	0.1/0.3	12.2	0.3/HM	0.52/2.7	
7	JU0426	5	49	NB	1.2/1.2	− 0.08/− 0.08	19.8	0.9/0.8	0.05/0.1	
8	JU0433	6	58	NB	0.7/1.0	0.15/0	11.7	0.7/0.7	0.15/0.15	
9	JU1850	6	68	NB, photophobia	0.4/0.3	0.4/0.52	0	0.4/0.3	0.4/0.52	
10	JU0752	7	43	Photophobia	0.9/1.0	0.05/0	8.0	0.2/0.7	0.7/0.15	
11	JU1817	7	26	NB	0.02/1.0	1.7/0	24.0	0.08/0.01	1.1/2.3	
12	JU1059	8	45	NB, decreased VA	1.2/1.5	− 0.08/− 0.18	6.2	0.9/1.2	0.05/− 0.08	
13	JU1248	9	5	NB	1.2/1.0	− 0.08/0	3.9	1.0/1.5	0/− 0.18	
14	JU1254	10	26	Distorted vision	1.5/0.5	− 0.18/0.3	5.6	1.2/0.6	− 0.08/0.22	MH in LE
15	JU1457	11	9	Decreased VA	1.2/0.07	− 0.08/1.15	2.6	0.2/0.3	0.7/0.52	CNV in BE
16	JU1458	11	9	No symptom	1.2/1.2	− 0.08/− 0.08	2.6	0.3/1.0	0.52/0	CNV in RE
17	JU1520	11	76	NB, decreased VA	HM/0.5	2.7/0.3	1.9	HM/0.6	2.7/0.22	
18	JU1681	12	13	Decreased VA	1.5/0.15	− 0.18/0.82	1.5	1.5/0.15	− 0.18/0.82	CNV in LE
19	JU1681b	12	11	No symptom	1.2/1.2	− 0.08/− 0.08	0	1.2/1.2	− 0.08/− 0.08	
20	JU1808	13	47	NB	0.6/0.6	0.22/0.22	8.3	0.5/0.6	0.3/0.22	
21	JU1877	13	42	NB	1.2/0.8	− 0.08/0.1	0	1.2/0.8	− 0.08/0.1	
22	JU1831	14	16	Decreased VA	0.01/1.2	2.30/− 0.08	0.2	0.01/1.2	2.3/-0.08	CNV in RE
23	JU1835	15	39	NB, photophobia	1.2/1.2	− 0.08/− 0.08	0.3	1.2/1.2	− 0.08/− 0.08	
24	JU1688	16	46	NB, Decreased VA	0.8/1.2	0.1/− 0.08	0	0.8/1.2	0.1/− 0.08	

*NB* night blindness, *VA* visual acuity, *BCVA* best-corrected visual acuity, *HM* hand motions, *RE* right eye, *LE* left eye, *BE* both eyes, *MH* macular hole, *CNV* choroidal neovasscularizetion.

The left eye (LE) of patient 1 had been enucleated due to ocular trauma. Our patients presented with a variety of chief complaints including night blindness only (9/24 patients, 37.5%), night blindness and photophobia (3/24 patients, 12.5%), night blindness and visual field defect (1/24 patient, 4.2%), night blindness and decreased visual acuity (3/24 patients, 12.5%), photophobia (2/24 patients, 8.3%), decreased visual acuity (3/24 patients, 12.5%), distorted vision (1/24 patient, 4.2%), and no symptoms (2/24 patient, 8.3%). The two patients (patients 16 and 19) without any symptoms, whose brothers were diagnosed with CHM, underwent ophthalmic examinations for diagnosis of CHM.

### Visual acuity outcome

**Figure 2:**
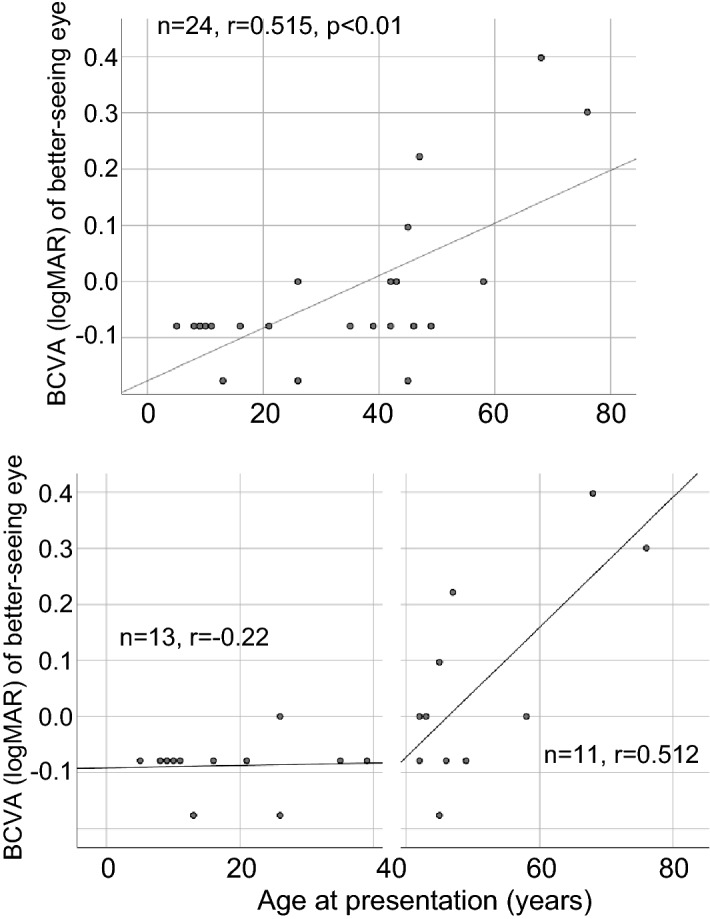
Scatter plots of the best-corrected visual acuity as a function of age. The best-corrected visual acuity (logMAR equivalent) of the better-seeing eye significantly declined as a function of age (n = 24, r = 0.515, p < 0.01). The segmented linear regression [≤ 40 years old (n = 13, r = − 0.22) and > 40 years old (n = 11, r = 0.512)] indicates that BCVA worsens particularly for > 40 years old.

**Figure 3:**
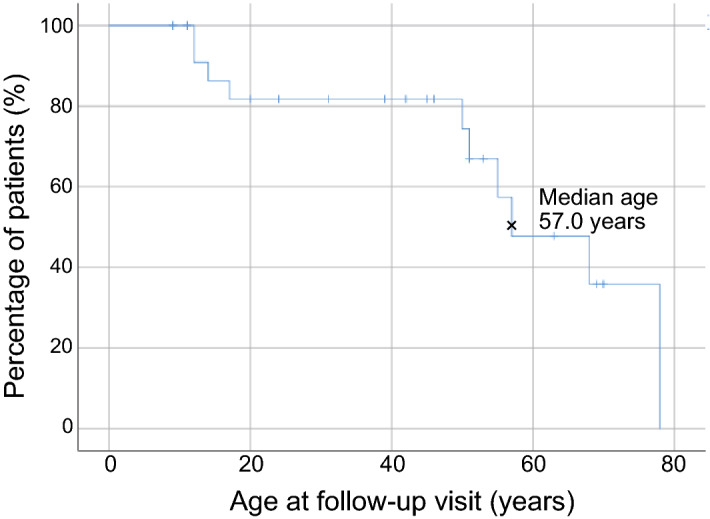
Kaplan–Meier survival curve. The Kaplan–Meier survival curve for a decimal BCVA equal of 0.5 (Snellen equivalent 20/40) or worse in the worse-seeing eye (n = 24) at follow up demonstrated that the median age of survival was 57.0 years.

As for visual acuity, there were differences in the logarithm of the minimum angle of resolution (logMAR) acuities at presentation between the right eye (RE) and LE in 7 patients due to sight-threatening retinal conditions such as macular hole (LE of patient 14^15^), choroidal neovascularization (CNV) [both eyes (BE) of patient 15, RE of patient 16, LE of patient 18], and severe chorioretinal atrophy (RE of patient 11, RE of patient 17, and RE of patient 22). Scatter plots of the decimal best-corrected visual acuity (BCVA) as a function of age were constructed. There was a significant correlation (n = 24, r = 0.515, p < 0.01) between BCVA (logMAR) of the better-seeing eye and age at presentation (Fig. 2a). We evaluated segmented linear regression [≤ 40 years old (n = 13, r = − 0.22) and > 40 years old (n = 11, r = 0.512)] because good BCVA was maintained until 40 years old (Fig. 2b). These results indicated that BCVA worsened with increasing age especially > 40 years old. Next, to predict visual acuity outcomes during the disease course, a Kaplan–Meier survival curve was plotted for decimal BCVA equal to 0.5 or worse in the worse-seeing eye (n = 24) at follow up, demonstrating that the median age of survival was 57.0 years (Fig. 3).

### Multimodal retinal imaging

**Figure 4:**
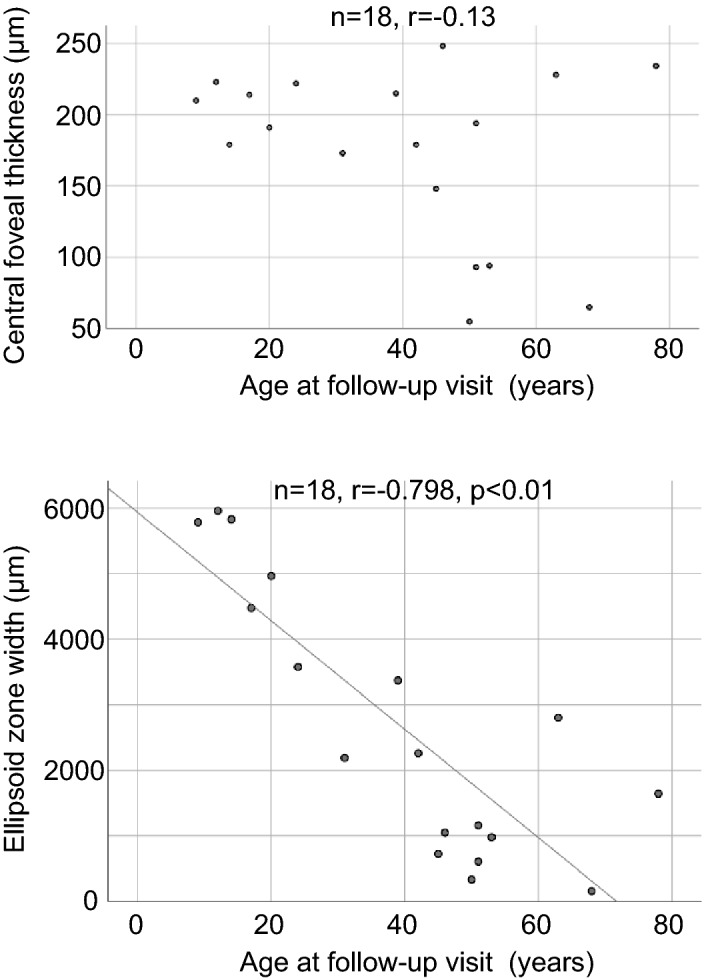
Scatter plots of optical coherence tomography findings of the better-seeing eye and age at follow up. No correlation between the central foveal thickness and age is found (n = 18, r = − 0.13). On the other hand, there is a significant negative correlation between the ellipsoid zone width and age (n = 18, r = − 0.798, p < 0.01).

Among 22 patients assessed by optical coherence tomography (OCT), 4 patients were excluded for unclear foveal ellipsoid zone (EZ) bilaterally (patients 6, 8, 20) and bilateral CNV (patient 15). We measured central foveal thickness (CFT) and the EZ width. We found no correlation (n = 18, r = − 0.13) between the CFT of the better-seeing eye and age at follow up (Fig. 4). On the other hand, there was a significant negative correlation (n = 18, r = − 0.798, p < 0.01) between the EZ width of the better-seeing eye and age at follow up (Fig. 4).

**Figure 5:**
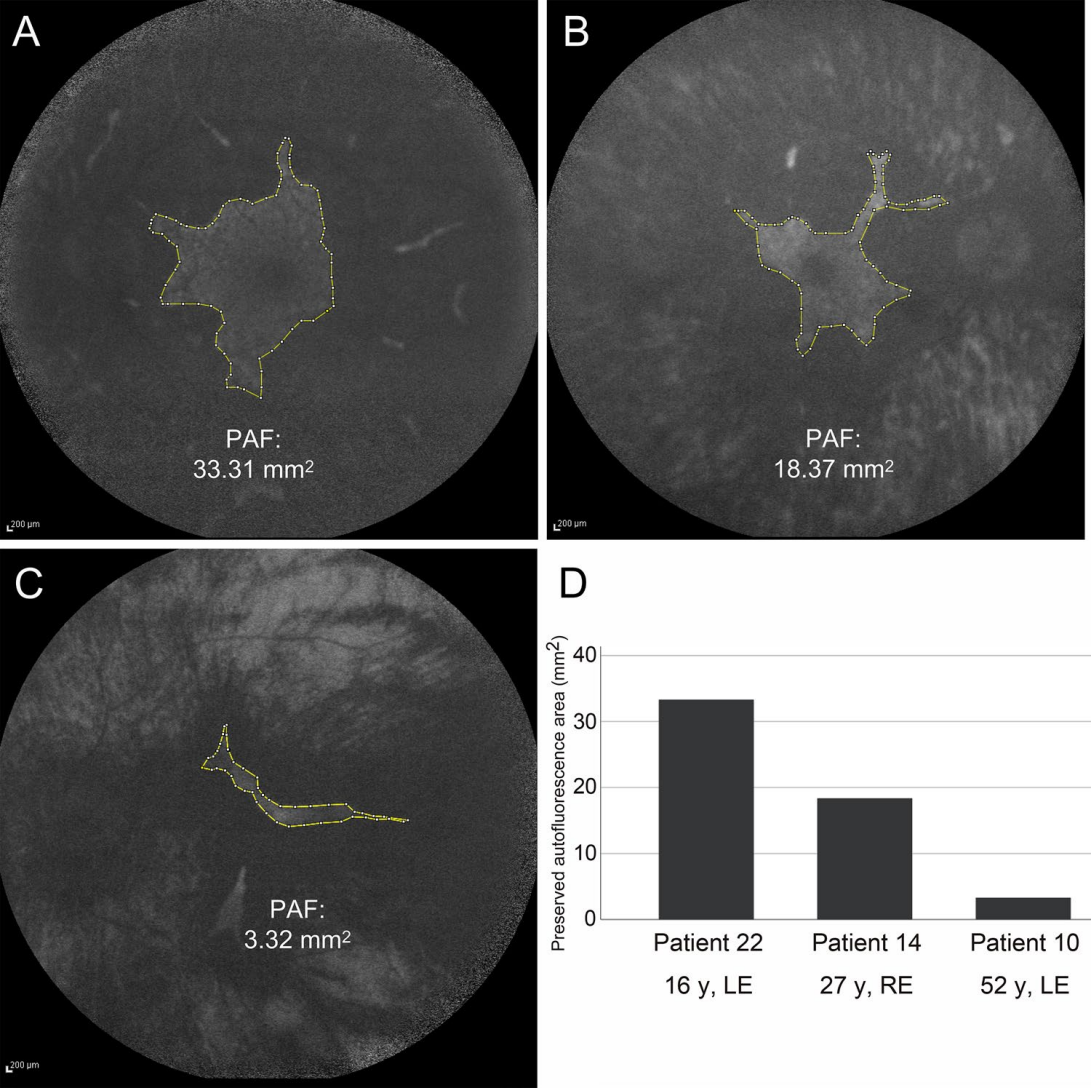
Fundus autofluorescence images. Fundus autofluorescence images are shown from the three representative patients. The preserved autofluorescence (PAF) area is measured from better-seeing eye of patients 22 **(A)**, 14 **(B)** and 10 **(C)** when they are 16 years, 27 years and 52 years of age, respectively. The PAF area in patient 10 is smaller than that in patients 22 and 14 **(D)**.

Fundus autofluorescence (FAF) images were shown from the three representative patients in Fig. 5. The preserved autofluorescence (PAF) area was measured from better-seeing eye of patients 22, 14 and 10 when they were 16 years, 27 years and 52 years of age, respectively. The PAF area in patient 10 was smaller than that in patients 14 and 22 (Fig. 5).

### Molecular genetic findings

A total of 14 different pathogenic variants including large deletions of *CHM* were identified in 16 families, but no pathogenic variant or deletion was found only in one family (family 6) (Fig. 1 and Table 2). Eight variants [c.315_318del (p.Ser105ArgfsTer20), c.616dupA (p.Thr206AsnfsTer17), c.646delA (p.Thr216LeufsTer16), c.757C > T (p.Arg253Ter), c.820-2A > T (splice site variant), c.1034C > G (p.Ser345Ter), c.1079delA (p.Asn360ThrfsTer49), and c.1649 T > C (p.Leu550Pro)] have been previously reported as causes of CHM^13,14,16–18^. However, 6 variants [c.49 + 5G > A (splice site variant), c.116 + 5G > A (splice site variant), c.527G > A, c.(529G > T; 530A > G);p.(Gly176Glu, Glu177Ter), c.1593 T > A (p.Tyr531Ter), a deletion of approximately 34.2 kb containing exon 2 (presence of exon 1 and exons 3–15), and a deletion of probably 5.0 mb containing the *CHM* gene] have not been reported in the Human Gene Mutation Database (HGMD) professional, ClinVar, or GnomAD. According to the American College of Medical Genetics and Genomics (ACMG) guidelines, p.Tyr531Ter and p.(Gly176Glu, Glu177Ter) were pathogenic and likely pathogenic, respectively, whereas both c.49 + 5G > A and c.116 + 5G > A were of uncertain significance (Table 2). As for c.49 + 5G > A, the different nucleotide changes (c.49 + 5G > C^19^ and c.49 + 5G > T^20^) at the c.49 + 5 position have been reported to be causes of CHM.

**Table 2:** Genetic findings of 24 patients with choroideremia.

Patient #	Patient ID	Family #	Nucleotide change	Protein change	HGMD	ClinVar/GnomAD	ACMG	References
1	JU0097	1	c.1593 T > A	p.Tyr531Ter	ND	ND	Pathogenic (PVS1,PM2,PP3)	This study
2	JU0141	2	c.820-2A > T	(Splicing change)	ND	ND	Pathogenic (PVS1,PM2,PP3)	Described^16^
3	JU0462	2						
4	JU0463	2						
5	JU0395	3	c.1079delA	p.Asn360ThrfsTer49	CD137317	ND	Pathogenic (PVS1,PM2,PP3)	Described^18^
6	JU0425	4	c.527G > A, c.(529G > T; 530A > G)	p.Gly176Glu, p.Glu177Ter	ND	ND	Likely pathogenic (PVS1,PM2)	This study
7	JU0426	5	Exon 2 deletion	(Gross deletion)	ND	Not applicable	Not applicable	This study
8	JU0433	6	*CHM* variant was not found					
9	JU1850	6						
10	JU0752	7	c.1034C > G	p.Ser345Ter	CM983733	ND	Pathogenic (PVS1,PM2,PP3)	Described^13^
11	JU1817	7						
12	JU1059	8	c.315_318del	p.Ser105ArgfsTer20	CD983792	ND	Pathogenic (PVS1,PM2,PP3,PP5)	Described^13^
13	JU1248	9	c.646delA	p.Thr216LeufsTer16	ND	ND	Likely pathogenic (PVS1,PM2)	Described^16^
14	JU1254	10	c.757C > T	p.Arg253Ter	CM994349	ND	Pathogenic (PVS1,PM2,PP3,PP5)	Described^14,15^
15	JU1457	11	c.616dupA	p.Thr206AsnfsTer17	CI1515670	ND	Likely pathogenic (PVS1,PM2)	Described^23^
16	JU1458	11						
17	JU1520	11						
18	JU1681	12	c.1649 T > C	p.Leu550Pro	CM093648	ND	Uncertain significance (PM2,PP3,PP5,BP1)	Described^17^
19	JU1681b	12	Not determined					
20	JU1808	13	c.616dupA	p.Thr206AsnfsTer17	CI1515670	ND	Likely pathogenic (PVS1,PM2)	Described^23^
21	JU1877	13						
22	JU1831	14	c.49 + 5G > A	(Splicing change)	ND	Likely pathogenic/ND	Uncertain significance (PM2,PP5,BP5)	This study
23	JU1835	15	Deletion of 5.0 Mb	(Gross deletion)	ND	Not applicable	Not applicable	This study
24	JU1688	16	c.116 + 5G > A	(Splicing change)	ND	ND	Uncertain significance (PM2,BP4)	This study

*ND* not described.

HGMD: the Human Gene Mutation Database (https://www.hgmd.org); ClinVar: https://www.ncbi.nlm.nih.gov/clinvar/; GnomAD: https://gnomad.broadinstitute.org/; ACMG: the American College of Medical Genetics and Genomics (VarSome, https://varsome.com).

**Figure 6:**
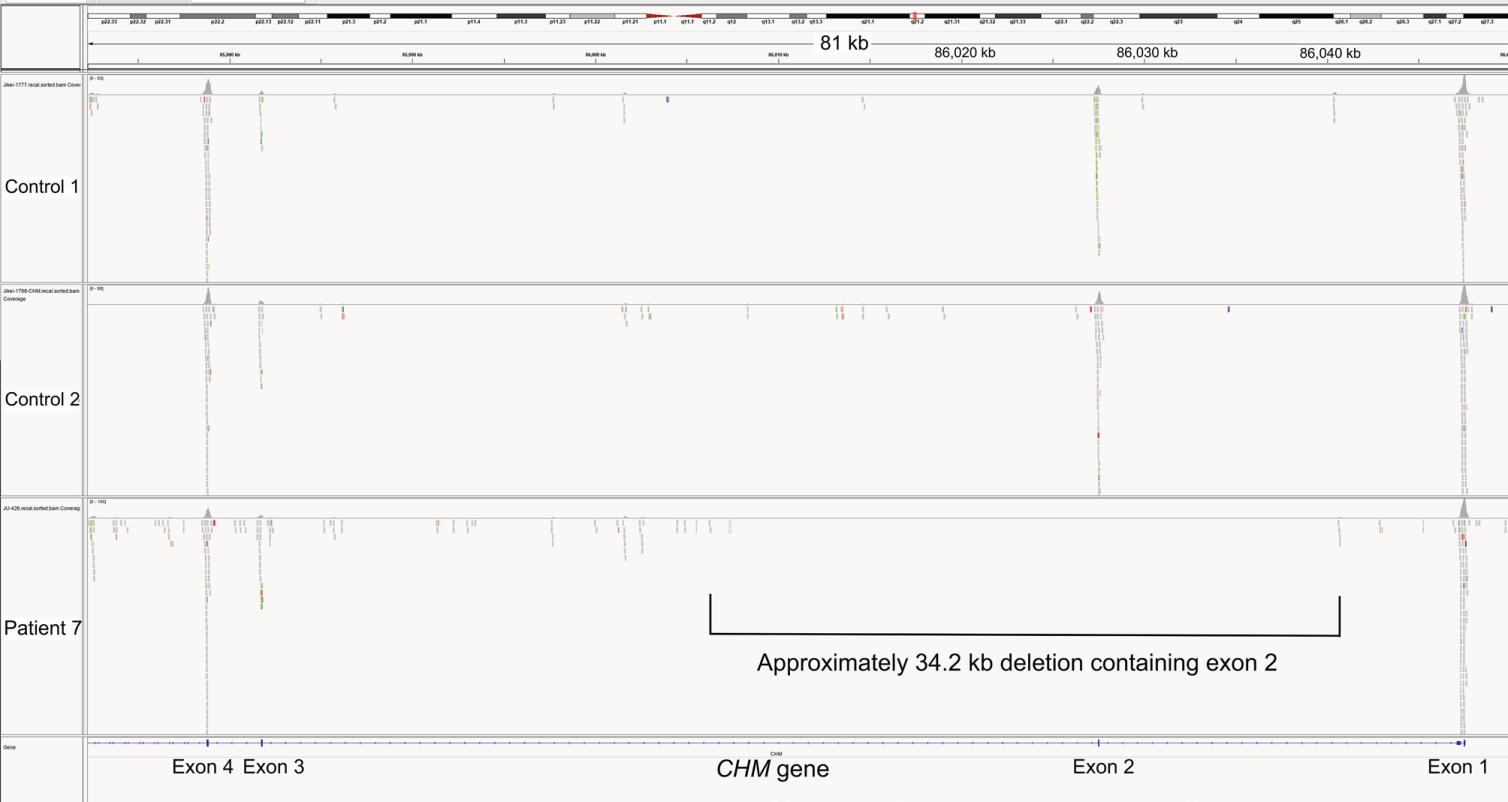
Whole-exome sequencing data of patient 7 and two controls. The Integrative Genomics Viewer visualization of exons 1–4 in the *CHM* gene indicated a deletion of approximately 34.2 kb containing exon 2 in patient 7.

We performed whole exome sequencing (WES) for patient 7 (family 5) due to the failure of polymerase chain reaction amplification of exon 2. However, all exons except for exon 2 were amplified for Sanger sequencing. The IGV demonstrated a deletion of approximately 34.2 kb containing exon 2, which was determined by comparison with two controls (Fig. 6). The presence of exon 1 and exons 3–15 was confirmed, consistent with the results of Sanger sequencing analysis. WES was also performed in patient 23 (family 15), demonstrating an extremely large deletion of probably 5.0 mb encompassing the entire *CHM* gene (Supplementary Figure S1) and other genes (*TGIF2LX*, *CPXCR1*, *KLHL4, DACH2*, *MIR1321*, *POF1B*, *SATL1*, *UBE2DNL*) in the vicinity of *CHM*.

In patients 8 and 9 (family 6), we performed whole genome sequencing (WGS) because no pathogenic *CHM* variant was found in both Sanger sequencing and WES. In addition, no pathogenic variant was found in both *RP2* and *RPGR* genes, which are responsible for X-linked retinitis pigmentosa. IGV revealed no decrease in read depth of any exons or introns in either patient (Supplementary Figure S2), indicating no obvious deletion region in the *CHM* gene. In addition, no rare variant was found in the promoter region. As a result, we could not find any pathogenic variant or deletion in the *CHM* gene, even though five male CHM patients were found in family 6.

## Discussion

In this study, we investigated the genetic spectrum of *CHM*, visual acuity outcomes and OCT findings in a single-center cohort of 24 Japanese patients (from 16 families) with CHM. Genetic analysis identified 14 different *CHM* variants or deletions, 6 of which were novel. The visual acuity outcomes revealed that BCVA significantly worsened with increasing age, and the Kaplan–Meier survival analysis suggested that a BCVA of 0.5 (Snellen equivalent 20/40) or better remains preserved until the fifties. Also, we found a significant negative correlation between the EZ width and age.

Previous studies have revealed that the majority of CHM patients harbor loss-of-function/null-type variants in the *CHM* gene^21,22^. In fact, our 13/14 (93%) variants were loss-of-function variants, including 4 nonsense [p.(Gly176Glu, Glu177Ter);family 4, p.Arg253Ter^14,15^;family 10, p.Ser345Ter^13^;family 7, and p.Tyr531Ter;family 1], 3 probable splicing (c.49 + 5G > A, c.116 + 5G > A, c.820-2A > T^16^), 4 small deletion/insertion (p.Ser105ArgfsTer20^13^ ;family 8, p.Thr206AsnfsTer17^23^;families 11 and 13, p.Thr216LeufsTer16^16^;family 9, and p.Asn360ThrfsTer49^18^;family 3), and 2 large deletion (families 5 and 15) variants (Table 2). Only one family (family 12) had a missense variant (p.Leu550Pro), which has been reported as the cause of CHM^17^. The Leu550 is well conserved, and the p.Leu550Pro variant leads to a conformational change by destabilization of β-structural elements^17^. Three variants (p.Ser105ArgfsTer20, p.Arg253Ter and p.Ser345Ter) are recurrent variants in the Japanese population^13,14^. Our genetic analysis demonstrated genetic variabilities of *CHM* in the Japanese population, although the same variant (p.Thr206AsnfsTer17) was identified in families 11 and 13, which were from the same prefecture.

Visual acuity is the most important parameter for the assessment of visual function. As for the relationship between BCVA and age, a cross-sectional study of 120 CHM patients, collected from 24 studies and/or case reports, showed that BCVA decreases very slowly until 50 years of age^24^. However, later vision loss becomes significantly higher^24^, which is supported by a subsequent cross-sectional study (n = 97) of the North American population^25^. Similarly, our correlation analysis revealed that BCVA significantly worsened with increasing age (Fig. 2a). In particular, the patients (> 40 years old) had a high rate of decline (Fig. 2b). Our results were consistent with the findings of the only previous Japanese cohort study of 15 CHM patients, showing that relatively good visual acuity is preserved until the forties^14^. Similar visual acuity outcomes, with a correlation of BCVA decline and age over 40 years, have been described even in different ethnic groups^26–29^. The natural disease course of visual acuity loss may be similar among CHM patients regardless of ethnicity.

An OCT study of 61 eyes from 39 CHM patients has revealed that the central retinal thickness was within normal limits until the 40 s, followed by significant thinning between 40 and 60 years of age^30^. Another study of 26 eyes from 13 CHM patients has demonstrated that no association was detected between CFT and age^31^. Our results also showed no correlation between the CFT and age (Fig. 4). While, there was a significant negative correlation between the EZ width and age (Fig. 4). Similar results have been previously described in CHM patients^12,32^. Previous studies of FAF analysis have revealed that a significant negative correlation is observed between age and the area of PAF^23,31,33^. The PAF area of patient 10 at 52 years old was smaller than that of patient 14 at 27 years old and patient 22 at 16 years old (Fig. 5). Thus, the age-dependent decrease in both EZ width and PAF area is likely to be associated with disease progression of CHM.

A previous study of *CHM* genotype and phenotype correlations found no significant difference in terms of visual acuity by variants involving the C-terminus and those occurring upstream^22^. Similar results were reported in a retrospective review study of CHM patients (n = 128), which found no apparent correlation between the *CHM* variant spectrum and decline in BCVA. The results showed that missense variants did not cause milder CHM phenotypes compared with entire deletions or other null variants^26^. Sanchez–Alcudia et al. reported the genotype and phenotype association of 36 patients with *CHM* variants and demonstrated that patients carrying complete deletions of *CHM* had an earlier onset of night blindness (9.6 ± 4.7 years old) compared with all patients (18 years old)^34^. However, patient 23, who had an entire deletion of the *CHM* gene (Supplementary Figure S1), presented with good visual acuity (Snellen equivalent 20/16) at 37 years of age (Table 1). Taken together, there is likely to be no correlation between the genotype of *CHM* and BCVA decline rate.

Until recently, no treatment was available for CHM. The first-in-human phase1/2 gene therapy trial was achieved in 2014 in which 6 CHM patients underwent vitrectomy accompanied with subfoveal injection of AAV2 containing CHM cDNA^8^. In the clinical trial, it was found that retinal sensitivities improved in two patients following the gene therapy. Subsequently, second stage phase 2 clinical trials (12–24 month follow up) using NSR-REP1 have been conducted in three different countries^10–12^. BCVA inclusion criteria (at baseline) in the treated eyes were equal to or worse than Snellen acuity 20/32 (logMAR 0.2) but better than or equal to Snellen acuity 20/200 (logMAR 1.0) in all three trials, demonstrating that visual acuity was maintained in the majority of patients and improved in some patients following gene therapy^10–12^. Based on the findings of these clinical trials, upcoming international phase 3 clinical trials of AAV2 gene therapy (NSR-REP1) are planned to evaluate the safety and efficiency of the therapy. For near future clinical trials, our data could play a pivotal role in understanding not only the natural history of visual acuity, but also the genetic spectrum of CHM patients in the Japanese population.

Our study had a few limitations, including a relatively small sample size (24 patients) and recruitment from a single center. Nevertheless, we found a variety of genetic variations: 14 different *CHM* variants in 15 families. In addition, our inclusion criteria might have had selection bias because some elderly CHM patients with total blindness, who cannot come to the hospital due to various reasons, might be excluded. Further studies with a larger sample size from multiple centers would give more strength to our visual acuity outcomes and OCT findings in disease progression.

In summary, we investigated the genetic spectrum of *CHM*, visual acuity outcomes and OCT findings in a single-center cohort of 24 Japanese CHM patients. Genetic analysis using Sanger sequencing and/or WES identified 6 novel variants/deletions in the *CHM* gene. The majority of *CHM* variants (14/15, 93%) were predicted to be loss-of-function/null-type variants in our Japanese families. The visual acuity outcomes revealed that BCVA significantly worsened with increasing age, especially in patients > 40 years old. The Kaplan–Meier survival analysis suggested that a BCVA of Snellen equivalent 20/40 or better remains preserved until the fifties. This was the largest cohort study to investigate visual acuity as a function of age in Japanese patients with *CHM* variants/deletions. Our results will be pivotal clinical data for upcoming phase 3 clinical trials of AAV2 gene therapy using NSR-REP1 to determine participants' eligibility as inclusion criteria for visual acuity.

## Materials and methods

### Participants

A single-center cohort of 24 CHM patients from 16 Japanese families was included in this study (Fig. 1). The medical records from The Jikei University Hospital were retrospectively reviewed including age, gender, chief complaint, visual acuity and OCT. The clinical diagnosis of CHM was made between 2002 and 2020, based on the characteristic ophthalmoscopic appearance, X-linked recessive transmission, and each carrier's ophthalmoscopy findings (when possible). We measured the BCVA at a 5-m distance using Landolt C charts at presentation and follow-up. Horizontal cross-setional retinal images (6.0 mm) were evaluated using spectral domain OCT (Cirrus HD-OCT, Carl Zeiss Meditec AG, Dublin, CA, USA) from all patients, except for patients 7 and 19, at follow-up visit. We measured the CFT and EZ width using a ruler within the OCT device. The CFT was difined as the distance between the vitreoretinal interface and the inner surface of the retinal pigment epithelium at the fovea, whereas the EZ width was difined as the horizontal linear distance between two locations. The Spectralis HRA2 (Heidelberg Engineering, Heidelberg, Germany) was used to obtain FAF images from patients 10, 14 and 22 as representative cases. The PAF area (mm^2^) was manually delineated and measured using the Image J software (National Institutes of Health, Bethesda, Maryland, USA). Genetic confirmation of the diagnosis was achieved in all 24 patients except for one patient (patient 19) who was a younger brother of patient 18 (family 12). Another patient (patient 14; family 10), who had a macular hole in his left eye, was previously reported^15^, and the phenotypes of female carriers in families 2, 9, and 11 were previously described clinically and genetically^16^.

### Molecular genetic study

Genomic DNA from leucocytes in venous blood samples was extracted using a Gentra Puregene Blood kit (Qiagen, Hilden, Germany) from the probands/patients (depicted by arrows in Fig. 1) and their family members. The exonic regions of the *CHM* gene were analyzed by Sanger sequencing^15,16^ and/or WES. WGS was performed in two patients (patients 8 and 9 in family 6) for whom no pathogenic variant nor deletion was detected by Sanger sequencing or WES. The details of WES and WGS methodologies were described previously^35–37^. After WES/WGS, called variants were filtered by allele frequencies (less than 0.01) using the Human Genetic Variation Database (HGVD; https://www.genome.med.kyoto-u.ac.jp/SnpDB/about.htm) among 271 genes listed in the RetNet database as of 2020.2.14 (https://sph.uth.edu/retnet/). Depth and coverage for the targeted areas were visualized and confirmed with the Integrative Genomics Viewer (https://www.broadinstitute.org/igv/)^37,38^. The novelty of *CHM* variants was evaluated using the HGMD professional as of 2020.1 (https://www.hgmd.org), ClinVar (https://www.ncbi.nlm.nih.gov/clinvar/) and GnomAD (https://gnomad.broadinstitute.org/). The pathogenicity of novel variants was determined by the VarSome (https://varsome.com/) according to the ACMG guidelines^39^. All *CHM* variants were validated by Sanger sequencing, and co-segregation was performed as much as possible. The *CHM* gene promoter region was analyzed by Sanger sequencing for the two patients (patients 8 and 9 in family 6) using a forward primer, 5′-ACTCAAATGGCGATAAGCACTG-3′ and a reverse primer, 5′-GAGCTACAGCATTCAGCCTGG-3′ as previously described^40^. We used the transcript sequence (NM_000390.3) of the *CHM* gene. The nomenclature of nucleotide and amino acid variants followed the conventions of the Human Genome Variation Society (HGVS) (https://varnomen.hgvs.org/).

The Database used in the study are publicly available.

### Statistical analysis

The mean, median, and range (min and max values) were used for assessment. Decimal BCVA was converted to the logMAR units for statistical analysis. BCVA of hand motions was converted to 2.7 logMAR units^41^. Spearman's rank correlation coefficient was used to evaluate the relationship between age and logMAR acuity, age and CFT, age and EZ width. P values < 0.01 were considered statistically significant. A Kaplan–Meier survival curve was plotted to estimate decimal BCVA 0.5 (Snellen equivalent 20/40) or worse in the disease course. Statistical and Kaplan–Meier survival analyses were performed using IBM SPSS Statistics version 26.0 (IBM Corp, Armonk, NY, USA).

### Ethics statement

The Institutional Review Boards of The Jikei University School of Medicine (approval number: 24-231 6997), National Hospital Organization Tokyo Medical Center (approval number: R14-050) and Nippon Medical School (approval number: 27-02) approved the protocol for this study. The protocol adhered to the tenets of the Declaration of Helsinki, with informed consent obtained from participants and/or their legal guardians.

## Supplementary information

Supplementary Information.
