# Supplementary material for: Genetic defects of CHM and visual acuity outcome in 24 choroideremia patients from 16 Japanese families
Source: Sci Rep. 2020 Sep 28;10:15883. doi: 10.1038/s41598-020-72623-1 (PMC7522719; doi:10.1038/s41598-020-72623-1)
Supplement: Supplementary file 1 — Supplementary Information. [file 41598_2020_72623_MOESM1_ESM.pdf]

Genetic defects of *CHM* and visual acuity outcome in 24 choroideremia patients from 16 Japanese families

Running title: *CHM* variants in Japanese choroideremia patients

Takaaki Hayashi [1,2], Shuhei Kameya [3], Kei Mizobuchi [2], Daiki Kubota [3], Sachiko Kikuchi [3], Kazutoshi Yoshitake [4], Atsushi Mizota [5], Akira Murakami [6], Takeshi Iwata [4], Tadashi Nakano [2]

[1]Department of Ophthalmology, Katsushika Medical Center, The Jikei University School of Medicine, Tokyo, Japan

[2]Department of Ophthalmology, The Jikei University School of Medicine, Tokyo, Japan

[3]Department of Ophthalmology, Nippon Medical School Chiba Hokusoh Hospital, Chiba, Japan

[4]Division of Molecular and Cellular Biology, National Institute of Sensory Organs, National Hospital Organization Tokyo Medical Center, Tokyo, Japan

[5]Department of Ophthalmology, Teikyo University School of Medicine, Tokyo, Japan

[6]Department of Ophthalmology, Juntendo University Faculty of Medicine, Tokyo, Japan

Corresponding author: Takaaki Hayashi, MD, PhD

Department of Ophthalmology, Katsushika Medical Center

The Jikei University School of Medicine

6-41-2 Aoto, Katsushika-ku, Tokyo 125-8506, Japan

Phone: +81-3-3603-2111, Fax: +81-3-3433-1936

E-mail: taka@jikei.ac.jp

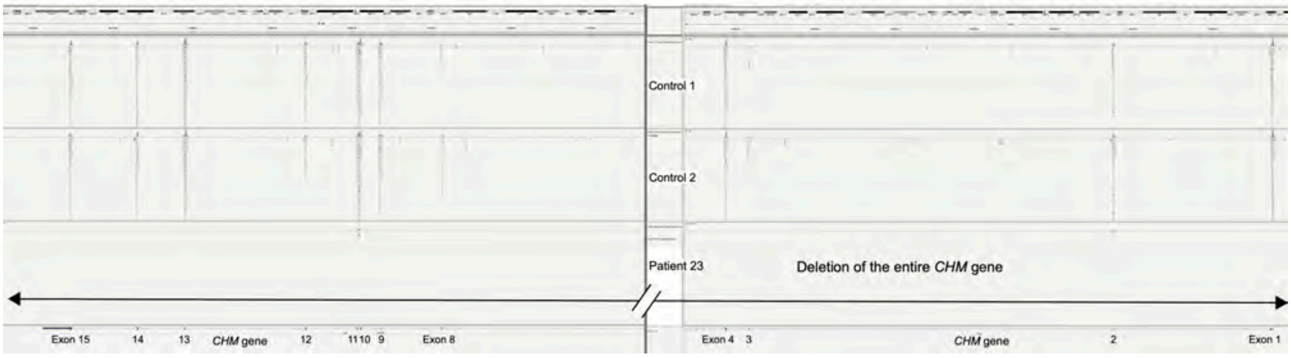

Supplementary Figure 1 Whole-exome sequencing data of patient 23 and two controls Whole-exome sequencing revealed an extremely large deletion of probably 5.0 mb encompassing the entire *CHM* gene and other genes (*TGIF2LX, CPXCR1, KLHL4, DACH2, MIR1321, POF1B, SATL1, UBE2DNL*) in the vicinity of *CHM*. The Integrative Genomics Viewer visualization of the *CHM* gene region demonstrated a deletion of the entire *CHM* gene (exons 1–15).

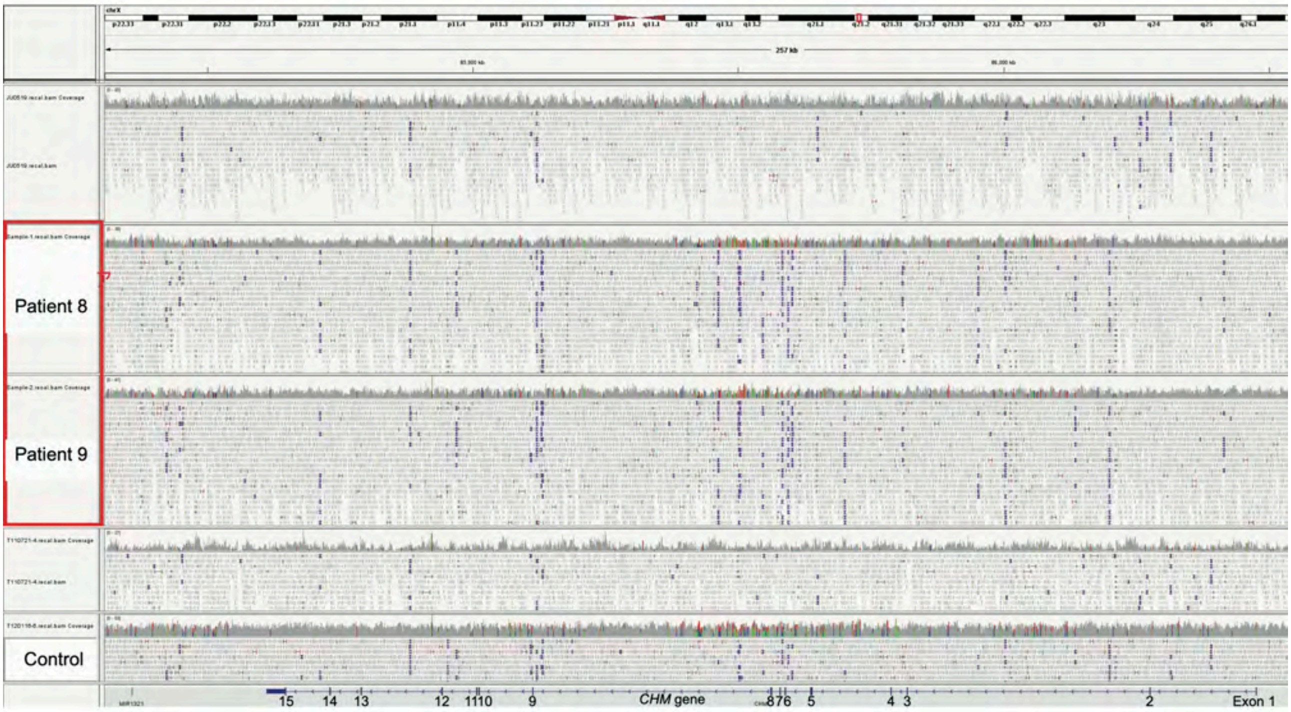

Supplementary Figure 2 Whole-genome sequencing (WGS) data of the *CHM* gene region for patients 8 and 9 and one control

The Integrative Genomics Viewer visualization revealed no decrease in read depth of any exons or introns in either patient, indicating no obvious deletion region in the *CHM* gene.
